# The “Frail” Brain Blood Barrier in Neurodegenerative Diseases: Role of Early Disruption of Endothelial Cell-to-Cell Connections

**DOI:** 10.3390/ijms19092693

**Published:** 2018-09-10

**Authors:** Jessica Maiuolo, Micaela Gliozzi, Vincenzo Musolino, Miriam Scicchitano, Cristina Carresi, Federica Scarano, Francesca Bosco, Saverio Nucera, Stefano Ruga, Maria Caterina Zito, Rocco Mollace, Ernesto Palma, Massimo Fini, Carolina Muscoli, Vincenzo Mollace

**Affiliations:** 1 IRC-FSH Department of Health Sciences, University “Magna Græcia” of Catanzaro, Campus Universitario di Germaneto, 88100 Catanzaro, Italy; jessicamaiuolo@virgilio.it (J.M.); micaela.gliozzi@gmail.com (M.G.); xabaras3@hotmail.com (V.M.); miriam.scicchitano@hotmail.it (M.S.); carresi@unicz.it (C.C.); federicascar87@gmail.com (F.S.); francescabosco@libero.it (F.B.); saverio.nucera@hotmail.it (S.N.); rugast1@gmail.com (S.R.); mariacaterina.zito@gmail.com (M.C.Z.); palma@unicz.it (E.P.); muscoli@unicz.it (C.M.); 2 Department of Medicine, Chair of Cardiology, University of Rome Tor Vergata, Via della Ricerca Scientifica, 00133 Rome, Italy; rocco.mollace@gmail.com; 3 IRCCS San Raffaele, Via di Valcannuta 247, 00133 Rome, Italy; massimo.fini@sanraffaele.it

**Keywords:** brain blood barrier, endothelial dysfunction, neurodegeneration

## Abstract

The main neurovascular unit of the Blood Brain Barrier (BBB) consists of a cellular component, which includes endothelial cells, astrocytes, pericytes, microglia, neurons, and oligodendrocytes as well as a non-cellular component resulting from the extracellular matrix. The endothelial cells are the major vital components of the BBB able to preserve the brain homeostasis. These cells are situated along the demarcation line between the bloodstream and the brain. Therefore, an alteration or the progressive disruption of the endothelial layer may clearly impair the brain homeostasis. The proper functioning of the brain endothelial cells is generally ensured by two elements: (1) the presence of junction proteins and (2) the preservation of a specific polarity involving an apical-luminal and a basolateral-abluminal membrane. This review intends to identify the molecular mechanisms underlying BBB function and their changes occurring in early stages of neurodegenerative processes in order to develop novel therapeutic strategies aimed to counteract neurodegenerative disorders.

## 1. Introduction

The Central Nervous System (CNS) is the most complex and highly organized system in the human body and its optimal functional state depends on the maintenance of stable biochemical conditions. In fact, to better perform their functions, neurons require a constant balance among various chemical (i.e., modulators and neurotransmitters) and electrical signals aimed to sustain the proper intra-cellular and inter-cellular communication. In particular, a constant intracellular and extracellular concentrations of ions (i.e., Na^+^, Ca^2+^, K^+^) and signaling molecules must be maintained in order to ensure the right amount of substrates to remove catabolites and to keep low concentrations of neurotoxic mediators. To this purpose, the blood-brain barrier (BBB) represents a physical and functional barrier able to provide all these functions, which separates the CNS from systemic circulation. 

The layer of endothelial cells characterizing brain capillaries represents a peculiar sequence of tightly sealed cell contacts that result in high trans-endothelial electrical resistance and low paracellular and transcellular permeability [1]. This particular architecture defines a complex, lipophilic, and highly selective barrier that protects the brain tissue and separates it from the bloodstream. Nevertheless, the BBB remains a highly dynamic structure specialized for the maintenance of brain homeostasis [2]. Basically, the BBB (1) ensures an adequate ion concentration, (2) prevents exotoxins and endotoxins uptake into the brain, (3) supports the normal neuronal and glial activity, and (4) controls the cell-mediated immune response against infectious agents. 

The development of the BBB is a multi-step process starting in the neuro-ectoderm with angiogenesis [3]. The endothelium of the BBB has specific proteins acting as transporters and receptors, which are responsible for the passage of metabolites, macronutrients, micronutrients, and junction proteins, which significantly limit their intercellular exchange. In addition, brain endothelium is characterized by the lack of fenestrae and a few transcytotic vesicles [4,5]. Moreover, the number of mitochondria is five or six times greater than other tissues of the human body since these organelles provide the energy needed for endothelial cells to maintain brain homeostasis [6]. 

Therefore, endothelial dysfunction leads to “frailty” of BBB, which is characterized by increased vascular permeability and is associated with an impaired ability to preserve brain tissue homeostasis. In turn, BBB breakdown promotes the infiltration of toxic blood-derived molecules, cells, and microbial agents in the brain parenchyma, which triggers inflammatory and immune responses and finally leads to neurodegeneration. 

In this paper, we will review physiological mechanisms underlying the endothelial cell tight connection and functioning in BBB and their impairment in the development of BBB “frailty” and neurodegenerative diseases. 

## 2. Junction Proteins of the Endothelial Cells

**Figure 1:**
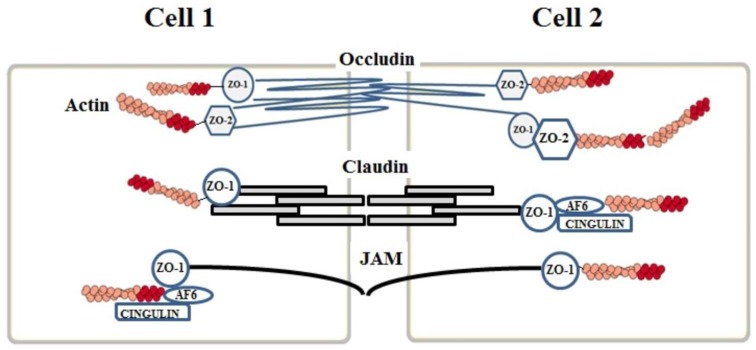
Molecular organization of the endothelial junctional complex in the blood-brain barrier (BBB).

Junction proteins involve “Tight Junctions (TJ),” “Adherens Junctions (AJ),” and “gap junctions.” Gap junctions are responsible for intercellular communication and can be lacking while TJ and AJ proteins control the permeability of the endothelium [7]. Figure 1 summarizes the localization of endothelial junction proteins and their potential role under physiological conditions. 

### 2.1. Tight Junctions Complex

The TJ complex is composed of three types of integral membrane proteins: (a) claudins, (b) occludins, and (c) junctional adhesion molecules (JAMs) to which some auxiliary cytoplasmic proteins such as ZO-1, ZO-2, ZO-3, and cingulins are added. The TJ complex is located in the apical region of the endothelial cells and its proteins act as an inter-endothelial demarcation between the apical and the basolateral side of the cell, which shows an asymmetric distribution of the membrane components [8]. 

The junctional proteins are located at the lateral membrane of the adjacent endothelial cells and, on the basis of their reciprocal interaction, are able to seal the intercellular gaps while the auxiliary cytoplasmic proteins establish a connection between the junction proteins and the intracellular cytoskeletal proteins. This is the pre-requisite to ensure the functional and structural integrity of the endothelium, which maintains BBB selective permeability and counteracts an excessive infiltration of immunocompetent cells in the brain tissue [8]. 

### 2.2. Claudins

The family of claudins is composed of 26 isoforms even though the most common ones are claudin 1, claudin 3, claudin 5, and claudin 12 [9]. The proteins belonging to this large family have a molecular weight ranging from 20 to 27 kDa and a composition ranging from 207 to 305 amino acids, which are organized in four transmembrane helical domains with the amino-terminus and the carboxy-terminus sequences located in the cytoplasm [10]. The extracellular loops of claudins interact with each other, which allows for a binding to those situated on the adjacent endothelial cells and leads to the formation of an intercellular “primary seal” [8]. The carboxy-terminus part of the claudins binds to the zonula occludens ZO-1, ZO-2, ZO-3 auxiliary cytoplasmic proteins. 

Human claudin-1 have 211 amino acids and its molecular weight amounts to 22,743 kDa. Moreover, it establishes homophilic and heterophilic interactions with other claudins (1, 3, and 5) belonging to the adjacent cells. Human claudin-3 consists of 220 amino acids and its molecular weight corresponds to 23,318 kDa. It is significantly involved in embryogenesis and postnatal development [11]. Evidence exists that claudin-3 expression tends to decrease inversely with age [12]. Human claudin-5 is made up of 218 amino acids and its molecular weight is 23,147 kDa. Its expression levels are about 100 times higher than other BBB claudins [13]. Experimental data showed that claudin-5 silencing induced an enhanced permeability of the BBB in the human brain [14]. On the other hand, the structure and function of claudin-5 are controlled by phosphorylation processes and its ubiquitinization on lys199 is mediated by a combined proteasomal and lysosomal pathway [15]. Moreover, recent evidence has shown that progressive inflammatory demyelination in cerebral adrenoleukodystrophy coincides with blood–brain barrier dysfunction, increased MMP9 expression, and changes in endothelial tight junction proteins including claudin-5 [16].

### 2.3. Occludins

Human occludins have a larger size than claudins. In fact, they consist of 522 amino acids and their molecular weight is 65 kDa. Seven occludin isoforms have been identified, which contain 11 tyrosine and 19 glycine residues responsible for the structural flexibility [17]. Occludins are the first proteins included in the TJ protein complex to be discovered [18] and are known as phosphoproteins. Their sequence has no affinity with claudins since they have four transmembrane helical domains with the carboxyl and the amino terminus facing the cytoplasm and two loops crossing the intercellular section. In contrast with claudins, the intracellular segments of occludins are in tight contact with the ZO proteins. 

Occludins possess multiple phosphorylation sites corresponding to the serine and threonine residues. Phosphorylation is directly involved both in the control of their combination with the cell membrane and in their function [19]. Moreover, occludins connect their intercellular extensions to those of the occludins located on the adjacent cells, which contributes to the closure of the gaps between endothelial cells. Recent evidence suggests a potential role of occludins as transducers of the signaling generated by cytokines in the inflammatory processes [20]. 

Lastly, cryptic ‘self’ tight junction antigens involving occludins can trigger an autoimmune response, which contribute to the neuro-inflammatory diseases such as Alzheimer’s disease and Parkinson’s pathology hallmarks [21].

### 2.4. Junctional Adhesion Molecules (JAMs)

The third class of proteins belonging to the TJ complex is represented by the Junctional Adhesion Molecules (JAMs) including three proteins (JAM-1, JAM-2, and JAM-3) of 40 kDa molecular weight. Recently, JAMs have been reported to be an immunoglobulin superfamily characterized by a single transmembrane domain with the intracellular section containing disulfide bridges. Apparently, JAMs proteins are not involved in the formation of the TJ complex but do take part in the assembly of the TJ components as well as in the adjustment of the complex responsible for endothelial polarization [22].

## 3. Auxiliary Cytoplasmic Proteins

In addition to the transmembrane TJ components, some auxiliary cytoplasmic proteins have been connected to the TJ complex and contribute in preserving the integrity of the brain endothelium. A special emphasis has been given to the members of the membrane-associate guanylate kinase (MAGUK) family, which are characterized by three PDZ, one SH3, and guanylkinase-like (GUK) domains. Among MAGUKs, the ZO proteins (ZO-1, ZO-2 and ZO-3) interact with the C-terminus of claudins and the JAM components interact through a PDZ domain [23,24] and with occludins through the GUK domain [23]. The ZO proteins play a crucial role in connecting the TJ complex to the actin cytoskeleton [25]. ZO-1 and ZO-2 are both phosphoproteins with a molecular weight of, respectively, 220 kDa and 160 kDa and have a high sequence homology even though it has been proven that only the dissociation of ZO-1 from the TJ complex may result in an increased permeability [26]. ZO-2, which interacts with ZO-1, seems to act as a transcription factor [27]. ZO-3 has a molecular weight of 130 kDa, but its function is unknown when compared to ZO-1 and ZO-2. In addition to ZOs, other auxiliary cytoplasmic proteins have been identified such as cingulin, AF-6, heterotrimeric G proteins (Gαi), and 7H6. These proteins bind to ZOs, JAM-1, and myosin in the cytoskeleton [28].

## 4. Adherens Junctions Complex (AJ)

The AJ complex basically includes cadherins, which contribute to the connection with the proteins of the AJ complex in the adjacent cells by using a calcium-dependent mechanism. Conversely, the intracellular domains bind to cytoskeletal structures [29]. The AJ complex performs three main functions: (1) connection of adjacent endothelial cells, (2) vascular growth and reshaping, and (3) promoter of the endothelial cell polarization process [7]. 

## 5. The Polarization Process

**Figure 2:**
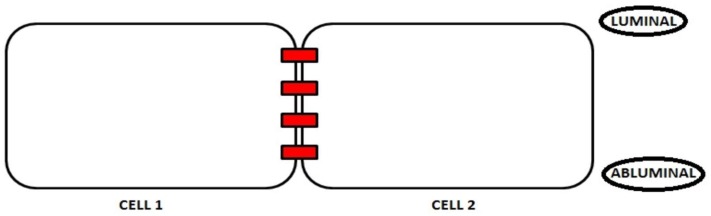
Localization of the luminal and abluminal sides of brain endothelial cells. The luminal and abluminal membrane domains are separated by tight junctions.

The polarity of the endothelial cells plays a key role in maintaining the integrity of the BBB, which is involved in counteracting the access of polar compounds, pathogens, and immune cells to the brain tissue [30]. Figure 2 shows localization of the luminal and abluminal sides of endothelial cells in the brain. Although the apical-basal polarity of the endothelial cells has been investigated for the first time during the brain angiogenesis step [31,32], the mechanisms underlying this process have not been elucidated. The release of the Vascular Endothelial Growth Factor (VEGF) by the neuronal precursors induce the endothelial cells to extend their filopodia and lamellipodia towards the direction of migration [33]. As the brain endothelium polarization process occurs in a limited area corresponding to the thickness of the cells, the apical-basal membrane localization of the proteins is difficult to establish [34]. Nevertheless, evidence has been collected that suggests a clear asymmetry exists in the composition of the brain endothelial membranes both in the protein and in the lipid content [35]. For example, the main transporters are responsible for the accumulation of the molecules in the brain and the active transport in both directions exclusively depend on their position in different membranes [36]. Moreover, the lipid distribution differs among apical-basal membranes. In fact, the apical-luminal side of the brain endothelium is coated with a glycocalyx composed of glycoproteins and proteoglycans, which are totally absent in the basolateral-abluminal side [37]. The latter, conversely, features many caveolae and micro-domains rich in sphingomyelin and glycosphingolipids [38]. 

The brain endothelium polarity is also crucial for the preservation of the BBB integrity. In fact, it controls the expression and function of the intercellular junction proteins [30]. In turn, endothelial junctions play a fundamental role for the preservation of polarity [39], which demonstrates that both factors cooperate to ensure the optimal functionality of the BBB. The intercellular junctions provide connection sites for the components, which underlies the cell polarity and, vice versa, the polarity process controls the expression and function of the intercellular junction proteins [30]. 

The endothelial polarity process is ensured by protein complexes such as Par, Crumbs, and Scribble, which are responsible for the polarity control [40]. The members of the Par complex, PAR-3, PAR-6, aPKC, and CDC42 are localized at the endothelial cell TJs through a direct link between PAR-3 and JAM proteins [41]. 

The Crumbs complex includes trans-membrane proteins oriented towards the TJs. The main members of this complex are PALS1, PATJ, which are involved in the reduction of the para-cellular permeability [42]. Scribble is composed by the DLG e SCRIB proteins and mediates the planar cell polarity and the migration of endothelial cells [43].

## 6. Junction Proteins and Neurodegeneration

Neurodegeneration is often characterized by an altered connection between TJs and AJs, which leads to a loss of the endothelial polarity and the impairment of the BBB integrity. In particular, the altered expression of Junction proteins can affect BBB in aging, in pathological states such as neurodegenerative diseases, and under dysfunction of physiological processes including an altered sleep-wake cycle and the equilibrium of gut microbiota. It has been demonstrated that numerous neurodegenerative diseases (i.e., stroke, multiple sclerosis, Parkinson′s disease, Alzheimer′s disease) and cancer as well as pathogenic infections and other pathological conditions, which are connected to the overproduction of both inflammatory cytokines and reactive oxygen species, can lead to an endothelial alteration at the BBB level and can be attributed to the disorganization and/or destruction of the junction proteins [44]. These events are followed by two main alterations: (1) growth of the passive diffusion of blood-derived substances causing the formation of edema and (2) massive infiltration of cells of the immune system through the BBB. 

**Figure 3:**
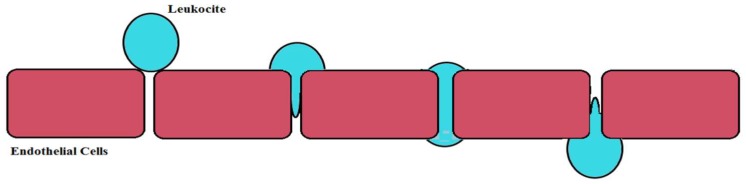
Loss of the blood-brain barrier (BBB) integrity facilitates pro-inflammatory and leukocytes infiltration.

Figure 3 shows that the loss of the blood-brain barrier (BBB) integrity facilitates pro-inflammatory and leukocytes infiltration. Post-mortem histopathological studies have shown clear alterations of the TJ proteins and micro-bleeds in the brain in line with aging [45]. 

The chronic reduction of sleep hours has been linked to a lower expression of the TJ complex proteins, a limited glucose uptake, and the resulting increase in the permeability to sodium. However, these altered TJ protein levels can be reverted by re-establishing the sleep-wake cycle [46]. 

An alteration of the gut microbiota may be associated with lower levels of claudin-5 and occludins as well as with an increased BBB permeability even though the related mechanisms require further investigation [47]. 

Despite growing evidence showing that the disassembly of the TJ and AJ complexes results in a higher BBB permeability, the alteration of the TJ complex is the main factor underlying the impairment of the endothelial cells. In particular, many mechanisms able to affect TJs are already known including the processes of phosphorylation in serine-threonine or tyrosine residues, the altered degradation, and the translocation of junction proteins as well as their down-regulation [19,48]. Dephosphorylated occludins were detected in brain endothelial cells and in an experimental autoimmune encephalomyelitis model that mimics multiple sclerosis [49] while a phosphorylation in threonine 207 of claudin-5 at the site of the carboxy-terminus altered BBB integrity [50]. 

Although protein kinase C (PKC) signaling has previously been related to the destruction of the BBB mostly during hypoxia [51], its involvement in BBB breakdown is still controversial. The activation of some isoforms due to hypoxic stimuli leads to an increased BBB permeability together with the re-localization of claudin-5, occludins, and ZO-1 [52]. Similarly, the activation of other PKC isoforms triggered by HIV/gp120 infection induces an increase of the intracellular calcium and the consequent BBB destruction [53]. Conversely, the activation of the nPKC isoform, after the accumulation of interleukin 25 (IL-25), results in the downregulation of occludins, claudin-5, and JAMs, which was followed by a reduction of the inflammation and preserves the BBB structure [54]. 

The alteration of the BBB permeability also involves the RhoA GTPase protein belonging to the G-protein family. In fact, it has been demonstrated that the activation of this protein causes cytoskeletal rearrangements during cell proliferation and migration besides the disorganization of the AJ complex [44]. Furthermore, the activation of the RhoA protein induces an increased permeability mediated by the phosphorylation in serine-threonine of occludins, claudin-5, and ZO-1 [55], which, in turn, triggers the migration of lymphocytes as clearly demonstrated in multiple sclerosis [56]. 

### 6.1. Brain Ischemia

In a mouse model of stroke and traumatic brain injury (TBI), the permeabilization of BBB develops following a “bi-phasic” opening of BBB in spite of an adequate reperfusion aimed to support the damaged neuronal tissue and to restore the normal bloodstream [57]. The development of BBB damage is characterized by a growing oxidative stress during 6 hours following after the injury and 48 to 72 h afterward by a massive infiltration of neutrophils due to the progression of the TJ complex alteration [58,59]. 

The release of reactive oxygen species (ROS) by neutrophils induces the other cells to produce a higher amount of cytokines, which attracts a greater concentration of leukocytes from the peripheral areas. Therefore, this prolongs the inflammatory cascade after an ischemic stroke and reduces BBB integrity [60]. 

This evidence was further confirmed by other studies showing structural alterations of the proteins belonging to the TJ complex, a higher transcytosis/endocytosis ratio, and a lower expression of occludins and ZO-1 after 48 to 58 hours from the ischemic insult. Conversely, during the earliest periods, a greater integrity of the TJ complex was observed [61].

### 6.2. Amyotrophic Lateral Sclerosis (ALS)

ALS is a neurodegenerative disease that involves a BBB alteration. In fact, in a mouse model of ALS, the motor neuron degeneration was characterized by a lower expression of occludins and ZO-1 at the molecular level [62], which was accompanied by a greater production of ROS responsible for the enhancement of the inflammatory response. In this context, the neurovascular alterations play a key role in the development of the ALS and the breakage of the BBB remarkably contributes to the progression of the disease [63]. 

### 6.3. Multiple Sclerosis

Multiple sclerosis is a neuro-inflammatory disease characterized by the infiltration of auto-reactive T-lymphocytes in the central nervous system and is the most widespread autoimmune disease in the Western world. Therefore, this disease clearly results from the BBB breakage and the resulting cell damage. The association of the dephosphorylation of occludins with the onset of various clinical symptoms has been proven at the molecular level [49], which suggests a close correlation between the higher BBB permeability and the alteration of endothelial junction proteins (occludin and ZO-1) [64]. Moreover, magnetic resonance imaging shows the diffusion of the gadolinium contrast medium through the TJ complex of the brain endothelium in the course of the active damages [65]. Similarly to multiple sclerosis, the increased infiltration of leukocytes leads to the inflammation of the CNS. In particular, it has been demonstrated that CD4+ lymphocytes produce interleukin 17 (IL-17) which, in turn, causes the destruction of the TJ complex in the brain endothelium and favors the progression of the disease [66].

### 6.4. Alzheimer’s Disease

Recently, it has been discovered that patients suffering from a mild or moderate Alzheimer’s disease featured BBB alterations [67]. The accumulation of peptide Aβ42 destroys the expression of ZO-1, which sensibly contributes to the increase in the BBB para-cellular permeability [68]. Furthermore, Aβ generates cytotoxicity in the endothelium with the resulting production and accumulation of a superoxide anion radical [69]. 

### 6.5. Viral and Bacterial Infections

The alteration of the BBB endothelium also originates from the diffusion of pathogens such as bacteria and viruses, which may affect the central nervous system. Bacteria cross BBB through different mechanisms. In particular, bacteria can pass the BBB, which colonizes lymphocytes or uses the leukocyte transendothelial migration and releases lipopolysaccharides (LPS), toxins, cytokines, or inflammatory molecules [70]. On the other hand, other bacteria such as Neisseria meningitides directly adhere to the endothelium through the interaction with multimeric structures called “type IV pili” [71]. Recent discoveries show that Streptococcus B infection leads to the up-regulation of the Snail1 protein in the brain endothelium, which represses the expression of TJ genes. At the same time, Snail1 reduces the levels of claudin-5, occludins, and ZO-1. This allows the bacterium to pass due to an altered TJ complex [72]. 

The virus-mediated neuronal alterations affect the BBB structure because of the destruction of the TJ complex. For example, the West Nile virus (WNV) leads to a significant loss of TJ proteins [73]. 

HIV patients have shown to develop neurological complications due to extensive neuronal damage such as cognitive and motor deficits [74]. Recent studies show that the onset of dementia and neurological disorders in AIDS patients are more evident when associated with an early abuse of drugs and medicines [75]. In this regard, some pre-clinical and clinical studies prove that the early or current abuse of psychostimulants may foster the diffusion of the HIV virus in the central nervous system, which is followed by the onset of neuro-AIDS disorders [76]. Cocaine or methamphetamine easily interact with the proteins of the external envelope of the HIV virus especially with protein gp120, which acts synergically against the BBB. In particular, oxidative stress induced by an infection inhibits the expression and affects the structure and the function of proteins composing the TJ complex [77,78]. 

## 7. BBB and Nutrients

The BBB endothelial cells are also involved in the transport and sorting of the nutrients by the blood to the central nervous system. The passage of metabolites is controlled by an elaborate highly restrictive junction complex acting between endothelial cells [79]. This is able to grant the continuity of the tissues, which reduces the random outflow of the compounds.

The transport type varies according to the chemical composition of the substance to be transported [80,81]. In fact, some mechanisms of passive and facilitated diffusion do not require any energy consumption while some kinds of active transport necessarily rely on an energy source. The highly lipophilic molecules smaller than 180 Da featuring less than 10 hydrogen bonds are able to cross the endothelial cell barrier through a passive diffusion process toward the concentration gradient. Similarly, the paracellular transport, which is exclusively passive, is guided by electrochemical and osmotic gradients [82]. Paracellular permeability is ensured by the balance resulting from both the contractile resistance of the endothelial cytoskeleton and the adhesive forces arising from the tight junctions between endothelial cells [83]. Under pathological conditions, an alteration of the adhesive properties of the molecules involved in the tight junction (TJs or AJs) or a reorganization of the cellular cytoskeleton occurs, which caused an impaired paracellular transport.

The facilitated transport mediated by carriers favoring the passage of small hydrophilic molecules [84] is recognized as the most important BBB transport system since it promotes the transport of nutrients from the blood to the brain [85]. 

Glucose is the main energy source for the brain tissue and its intake is catalyzed by the facilitated glucose transporters (GLUTs, SLC2As) and the sodium/glucose cotransporters (SGLTs, SLC5A). Among them, the most common transporter in mammals is GLUT1, which is expressed both in the luminal and in the abluminal endothelial membrane [86]. The functionality of these transporters results in insufficient levels of glucose with the consequent impairment of its homeostasis [87]. 

The brain energy level is also ensured by creatine and choline. Creatine is responsible for a higher production of ATP and its transport in the brain is mediated by the specific SLC6A8 sodium and chlorine dependent transporter [88]. Choline acts as a precursor for the neurotransmitter acetylcholine, which contributes to the formation of phosphatidylcholine and sphingomyelin and its intake is mediated by the choline transporter OCT2 [89]. 

The transport of amino acid across the BBB is catalyzed by facilitated diffusion carriers. Among them, specialized proteins (SLCs) are able to promote the transport of glutamate, aspartate, GABA, and glycine [90,91]. 

Furthermore, SLC carriers (ENTs, SLC29A, CNTs, SLC28A) are responsible for the transport of nucleosides (cytidine, uridine, adenosine, guanosine, thymidine, and inosine), nucleoside triphosphate (ATP, GTP, CTP, UTP), nucleobases (adenine, guanine, uracil, thymine, cytosine) [86], water-soluble vitamins, i.e., thiamine, biotin, folates and ascorbic acid [92] as well as essential metals acting as enzyme cofactors (Cu ^2+^, Zn 2^+^, Mn^2+^, Fe ^2+^, Cd^2+^, Co^2+^, and Ni^2+^) [93]. 

Lastly, the water passage must be assessed as well since its control is crucial for the preservation of the ionic concentration and the proper brain and BBB functioning [94,95]. The channels involved in the transport of water are membrane proteins, which are assembled in a specific pore named Aquaporins (AQP) whose main isoform is AQP4. This is abundantly expressed in the brain and at the BBB level [96]. 

The first form of the active transport across the BBB involves the ABC transporters (ATP-binding cassette) which, through the hydrolysis of the ATP molecule used as an energy source, ensure the entry and exit of many organic compounds through the membranes [97,98]. The superfamily of ABC transporters includes seven A-G subfamilies of which B, C, and G are involved in the efflux of xenobiotics since these subfamilies of transporters are expressed at the BBB as well as in the excretory system [99,100]. 

Some ABC subfamilies are involved in the transport of lipids, proteins, metals, and in drug resistance processes [101]. Recent studies have highlighted a specific correlation between the intake of some nutraceuticals and the ABC transporters. In fact, many plant products can inhibit the active efflux of ABC pumps, which reduces the excretion of several drugs and increases their absorption and bioavailability [102,103]. 

The last type of active transport examined in this study is the transcytosis, which is based on the bidirectional transport of clathrin-coated vesicles and contains nutrients and the relating carriers as well as ligand-receptor complexes, membrane proteins, bacteria, and viruses [104,105]. 

## 8. Nutrient Transport and Neurodegeneration

The alteration of the described transport mechanisms across BBB have detrimental effects on neurons, which lead to the onset of numerous neurodegenerative diseases [106]. 

For example, major disorders including epilepsy, hypotonia, spasticity, ataxia, and cognitive disorders have been associated with GLUT1 impairment [107,108]. Moreover, a reduced glucose transporter phosphorylation has been detected in different cerebral areas of patients affected by Alzheimer’s disease [109]. 

Many experimental mouse models presenting dysfunctional excitatory amino acid transporters at the BBB showed a 50% mortality rate of the animals involved in the study [110]. Patients affected by Parkinson’s disease revealed reduced valine, leucine, and isoleucine (amino acids) values into the cerebrospinal fluid [111]. 

The alteration of the SLC transporter of thiamine has been related to encephalopathy, dysarthria, dysphagia, stiffness, confused state, and even death. Moreover, the early onset of the Leigh syndrome together with progressive neurodegenerative phenomena and subsequent SLC transporter dysfunction have been detected [112]. 

A decrease in the iron levels caused by a defect in metal transport in the brain results in cognitive and motor disorders while its accumulation can induce oxidative stress, which induces the onset of chronic neurodegenerative diseases such as Parkinson’s disease, Huntington’s disease, and Alzheimer’s disease [113]. A mutation of the ATP7A copper transporter leads to a copper deficiency inducing neurological degeneration and growth retardation [114]. Diseases such as dystonia, cognitive impairment, Alzheimer’s disease, and Parkinson’s disease have been associated with copper accumulation in the brain [115].

The expression of the ABC transporters is largely reduced in the presence of some neurological diseases such as Alzheimer’s disease, Parkinson’s disease, epilepsy, and amyotrophic lateral sclerosis [116,117,118]. Moreover, the ABC transporters play a key role in many genetic disorders. In particular, the mutation of the ABCA subfamily gene reduced the HDL formation, which favors the onset of Tangier disease—an autosomal recessive disorder characterized by the lack of high density lipoproteins—and the resulting accumulation of cholesteryl esters [119]. 

Lastly, an alteration of transcytosis at the BBB endothelium also causes evident neurological impairments. In fact, there is a correlation between the alteration of transcytosis and ischemia-related brain damage [61,120,121]. Apparently, there is no connection between a lower transcytosis rate and the alteration of the endothelial junctions [122], but rather it is likely linked to an altered formation of the vesicle coating. The lack of this coating is observed in Alzheimer’s disease and multiple sclerosis [79]. Clathrin-coated vesicles are mainly involved in the transport of ligand-receptor complexes including those that carry LDLs and iron-transferrin towards the brain [123]. Compared to clathrins, caveolae are even more engaged in the impairment of transcytosis in the case of diseases affecting the BBB such as severe strokes and ischemia [84]. Uncoated vesicles have also been detected in patients suffering from Alzheimer’s disease and multiple sclerosis [124].

## 9. Conclusions

Endothelial cells are the main component of the blood-brain barrier and their compromised function has been associated with numerous diseases of the CNS including neurodegenerative and neuro-inflammatory disorders. The main mechanisms that may reduce the integrity of the BBB are ones that disrupt AJ and TJ complexes such as through inflammatory processes and a dysfunction in transport systems. As a consequence, the identification of mechanisms underlying the impairment of endothelial balance represents a priority for the development of new therapeutic strategies aimed to prevent BBB breakdown and the development of several neurodegenerative diseases.
